# Atrophic Progressive Myopathy

**Published:** 1886-08

**Authors:** 


### Atrophic Progressive Myopathy.

The nature of progressive muscular atrophy of infants has usually been regarded as the same as that of the adult. But Duchenne observed that the muscular atrophy of infancy began in the face, and therein differed markedly from the adult malady. MM. Landouzy and Dejerine, two well known French neurologists, now maintain the distinctive nature of the infantile affection. They do not regard it as a simple variety of the classic muscular atrophy, but assert dogmatically that the central nervous system shows no signs of disease. It is important to insist on the frequency with which hereditary antecedents may be obtained in the infantile disease; it runs in families, as Duchenne fully showed. The heredity may be direct or collateral. Most often the affection commences during the second infancy, but it may be delayed till adult age, or even till old age. It starts in the facial muscles usually, and but rarely in the upper limb. The atrophy of the facial muscles is visible as a deformity in the mouth, the lower lip falling, and both lips looking fuller than natural. Sometimes the orbicularis palpebrarum is so wasted as to cause genuine lagophthalmia. The masticators, the ocular muscles, the tongue, pharynx, and soft palate, are always respected by the disease. At the end of a certain time the atrophy appears in other muscles of the body, and is gradually generalized. The wasting is never rapid, but always insidious, and there are no pains or paralytic troubles. It not unfrequently invades the following muscles: trapezius, rhomboidei, deltoid biceps, triceps, long supinator, and the pectorals. The atrophy invades homologous muscles, and is symmetrical. The lower limbs are affected later, and the sacrolumbar masses also. A negative symptom of much importance is the *absence* of fibrillary contractions in the muscles; another is the absence of false hypertrophy; and a third is the preservation of the voluntary contraction with the absence of the reaction of degeneration. Erb's juvenile form of progressive muscular atrophy closely simulates that described as a myopathy by MM. Landouzy and Dejerine, but the facial muscles are not affected, and there is some false hypertrophy, though there are no fibrillar contractions and no reaction of degeneration. The most important fact is the absence of changes in the central nervous system.

---

THE medical profession of the United States numbers more than 77,000 members; the dental, a little more than 12,000.

